# Thermomechanical Material Characterization of Polyethylene Terephthalate Glycol with 30% Carbon Fiber for Large-Format Additive Manufacturing of Polymer Structures

**DOI:** 10.3390/polym16131913

**Published:** 2024-07-04

**Authors:** Katie A. Martin, Guillermo A. Riveros, Travis L. Thornell, Zackery B. McClelland, Elton L. Freeman, James T. Stinson

**Affiliations:** 1 Geotechnical and Structures Laboratory (GSL) at the US Army Corps of Engineers (USACE) Engineer Research and Development Center (ERDC), 3909 Halls Ferry Rd., Vicksburg, MS 39180, USA; katie.a.martin@usace.army.mil (K.A.M.); travis.l.thornell@usace.army.mil (T.L.T.); zackery.b.mcclelland@usace.army.mil (Z.B.M.); 2 Information Technology Laboratory (ITL) at the US Army Corps of Engineers (USACE) Engineer Research and Development Center (ERDC), 3909 Halls Ferry Rd., Vicksburg, MS 39180, USA; elton.l.freeman@usace.army.mil (E.L.F.); james.t.stinson@usace.army.mil (J.T.S.)

**Keywords:** large-format additive manufacturing (LFAM), additive manufacturing (AM), composites, polyethylene terephthalate glycol (PETG), carbon fiber (CF), thermomechanical characterization, bead-layer-print

## Abstract

Large-format additive manufacturing (LFAM) is used to print large-scale polymer structures. Understanding the thermal and mechanical properties of polymers suitable for large-scale extrusion is needed for design and production capabilities. An in-house-built LFAM printer was used to print polyethylene terephthalate glycol with 30% carbon fiber (PETG CF30%) samples for thermomechanical characterization. Thermogravimetric analysis (TGA) shows that the samples were 30% carbon fiber by weight. X-ray microscopy (XRM) and porosity studies find 25% voids/volume for undried material and 1.63% voids/volume for dry material. Differential scanning calorimetry (DSC) shows a glass transition temperature (T_g_) of 66 °C, while dynamic mechanical analysis (DMA) found T_g_ as 82 °C. The rheology indicated that PETG CF30% is a good printing material at 220–250 °C. Bending experiments show an average of 48.5 MPa for flexure strength, while tensile experiments found an average tensile strength of 25.0 MPa at room temperature. Comparison with 3D-printed PLA and PETG from the literature demonstrated that LFAM-printed PETG CF30% had a comparative high Young’s modulus and had similar tensile strength. For design purposes, prints from LFAM should consider both material choice and print parameters, especially when considering large layer heights.

## 1. Introduction

Additive manufacturing is defined as “a process of joining bulk raw materials to make parts from 3D model data, usually layer upon layer, as opposed to subtractive manufacturing and formative methodologies” [1]. Fused deposition modeling (FDM), or fused filament fabrication (FFF), is a type of 3D printing that extrudes molten plastic through a heated nozzle as the printer head moves on a pre-calculated path, building a part layer by layer from the cooling material [2,3,4]. Three-D printing can rapidly manufacture customizable parts with a wide variety of thermoplastic materials, including carbon fiber composites [5], with the low cost due to effective material usage and quick manufacturing time cycle [2], opening application and usage potential. There is a wide variety of large-format 3D printers, with build volumes varying from 372 m^3^ (Masterprint 3X—Ingersoll Machine Tools, Inc., Rockford, IL, USA) [6] and 35.5 m^3^ (BAAM—Cincinnati Labs, Harrison, Ohio, USA) to 0.48 m^3^ (German RepRap X1000, Feldkirchen, Germany) [7]. Large printers use pellets instead of filament to keep up with the required large material output [7]. Printing with pellets is sometimes referred to as fused granular fabrication (FGF) [8]. The high-volume extrusion allows for rapid manufacture of large-scale parts within a few days and significantly reduces the time needed to print medium-sized parts [6].

Shrinkage is a common concern in 3D printing [7,9]. Shrinkage is defined as the difference between expected and final dimension over the expected dimension [10] or, more specifically for 3D printing, the dimensional difference between the initial print conditions and the condition when the print is complete and fully cooled [4]. Shrinkage is caused by internal stresses from thermal gradients [7] and occurs as material cools from a high temperature [10]. Shrinkage becomes more likely as part size increases [7]. Shrinkage is predominately driven by the coefficient of thermal expansion, where carbon-fiber-reinforced composites and amorphous polymers have lower coefficients of thermal expansion when compared to the matrix material or crystalline polymers, respectively [10]. A low coefficient of thermal expansion is desirable for 3D printing [4].

FFF part strength is dependent on porosity (also referred to as void content), strength of bonding between beads, and material properties [11]. Generally, FFF printed parts have less strength than traditionally manufactured parts [3] because (1) the void space formed during the printing process results in stress concentrations and less bulk material per cross-sectional area and (2) the rapid cooling of beads limits polymer entanglements [3,4,12,13]. Higher print temperatures result in more diffusion of polymer chains and therefore a greater entanglement fraction, resulting in increased mechanical strength at the weld region [14]. Bonding between beads depends on the temperature, viscosity, and surface energy, while neck development between printed beads depends on viscosity, thermal conductivity, and higher temperatures [12]. Printing parameters that affect the development of void space include nozzle temperature, print bed temperature, printing speed, layer height, infill density and pattern, and position on the *z*-axis [3]. Post-processing such as annealing can affect a material’s crystallinity, while a lack of crystallinity after annealing indicates that it is amorphous [15].

Polyethylene terephthalate glycol (PETG) [5] is a common 3D-printing feedstock [4,5,9]. According to an internal investigation by the Navy, polylactic acid (PLA) [16] and acrylonitrile-butadiene-styrene (ABS) [16] produced damaging, poisonous, and cancerous fumes when they caught fire, while PETG did not, making PETG a better option for printing Navy vessels out of thermoplastic polymers. PETG is an amorphous material, so it does not have a melting temperature [4,6] and has good tensile strength, ductility, and durability, high chemical resistance, and high thermal stability [17].

FFF-printed parts are generally weaker than traditionally manufactured parts [3], so fillers such as fiber are added to increase mechanical strength [18]. Research has investigated the effect of different fillers such as continuous carbon fiber with epoxy [19], graphene [20], and glass fiber [6] on interlaminar strength [19], mechanical properties [20], and thermomechanical properties [6], respectively. Because carbon fiber (CF) has high tensile strength, it is used as a reinforcement material in polymer composites [16]. The addition of carbon fiber also reduces a polymer’s shrinkage [10], which is desired in LFAM. Carbon fiber can either be continuous or short-cut, in which short-cut fibers are mixed into the polymer matrix [18]. Given the increased strength from the CF and the good material properties from PETG, PETG CF30% was selected as the material to investigate.

Researchers have studied the effect of CF additive on traditionally manufactured and printed samples. Quintana et al. (2022) looked at the effect of fiber orientation on thermal properties of fiber-filled printed material for large prints (layer height of 5.08 mm). Adding glass fiber to PETG decreased the T_g_ and the coefficient of thermal expansion was non-isotropic within the printed bead, likely due to a difference of fiber alignment in the bead [6]. Kichloo et al. (2022) compared the flexural strength of 3D-printed PETG and CF-reinforced PETG (20 wt.%) at different print parameters (layer heights of 0.1 mm, 0.14 mm, and 0.18 mm). Kichloo et al. (2022) did not find the addition of CF to improve strength across all print parameters; instead, CF improved tensile strength at lower layer heights and improved flexural strength at higher layer heights [2]. Kováčová et al. (2020) compared hot-pressed PETG with different percentages of CF from 0% to 20%. Adding 20% CF to PETG resulted in increased tensile strength and increased Young’s modulus by 124%. Kováčová et al. (2020) determined that the carbon fiber had limited effect on the thermogravimetric properties, with the onset degradation temperature ranging from 397.2 °C to 402.1 °C, with a range of carbon fiber content from 0% CF to 20% CF. Increasing amounts of carbon fiber filler resulted in significantly lower coefficients of expansion, with PETG having a value of 60.12 × 10^−6^ K^−1^ and PETG CF20% having a value of 28.79 10^−6^ K^−1^, where a low value of thermal expansion results in decreased shrinkage during printing [9].

Fiber orientation is known to affect tensile strength. Short-cut fibers align during the printing process, resulting in anisotropy [18]. Compton and Lewis (2014) developed and 3D-printed a composite epoxy ink with silicon carbide whiskers and carbon fiber. Composite samples exhibited higher strength when tested in the same direction as the print direction due to carbon fiber alignment during printing [21]. Quintana et al. (2022) found that glass fiber and carbon fiber tended to align in the print direction for LFAM, which affected thermal behavior [6].

While there is information in the literature on FFF and the thermomechanical properties of 3D-printed PETG CF, there is limited research on thermomechanical properties of PETG CF30% printed with LFAM, where the difference in bead size and temperature profile may affect part strength. For LFAM to be used for production, an understanding of the difference in strength and thermomechanical properties as a result of LFAM should be developed. The work below studies the thermomechanical behavior of printed (large-scale), unprinted, dried, and undried PETG CF30%, with emphasis placed on practical implications for large-scale 3D printing (layer height 2 mm).

## 2. Materials and Methods

### 2.1. The High Output Research Printer (THOR)

**Figure 1:**
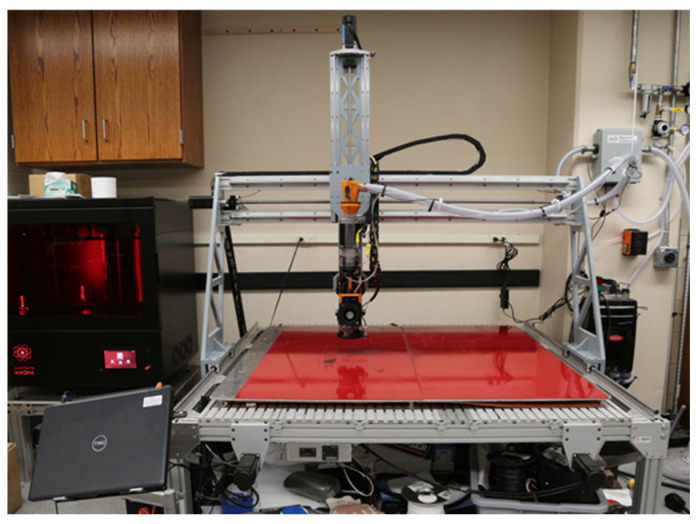
The High-Output Research Printer (THOR).

The High-Output Research Printer (THOR), shown in Figure 1, is a LFAM pellet-fed 3D printer system built in-house by the Geotechnical and Structures Lab at the US Army Corps of Engineers (USACE) Engineer Research and Development Center (ERDC) in Vicksburg, MS, USA from a Millright CNC Power Route XL Assembled (Millright, Leesburg, GA, USA) machine and a MDPH2 extruder (Massive Dimension, Barre, VT, USA). The approximate build area is 48″ × 48″ × 20″. A Prusa Slicer (version 2.6.1) with a custom print bed model was the slicer and Mach4 software (version 4.2.0.4612) was the printer operating system. The print bed and nozzle temperatures were controlled separately from the GCODE using PID controllers.

### 2.2. Print Material

Electrafil PETG 1711 3DP (PETG CF30%) in pellet form from Techmer PM Polymer Modifiers (Techmer PM, Clinton, TN, USA) was used for printing and materials characterization. Pellets were determined to have 30% carbon fiber by weight as described in Section 2.2.1. Pellets were dried in a Dri-Air Model HPD-4 RH-150 Drier (Dri-Air Industries, East Windsor, CT, USA) for about 6 h at 60–66 °C (140–150 °F).

#### 2.2.1. TGA

TGA was used to determine the carbon fiber content of undried PETG CF by comparing it with blue PETG filament (MatterHackers, Lake Forest, CA, USA). A TA Instruments TGA 550 (TA Instruments, New Castle, DE, USA) ran one sample of each material, and TA Instruments TRIOS software (version 5.1.1) was used for data collection and analysis. The samples were run in nitrogen at a rate of 10 °C/min to 1000 °C. The approximate amount of carbon fiber was determined by subtracting the percentage weight of the regular PETG from the PETG CF. The degradation onset temperature for both materials was determined using TRIOS software.

### 2.3. Sample Printing and Preparation

**Figure 2:**
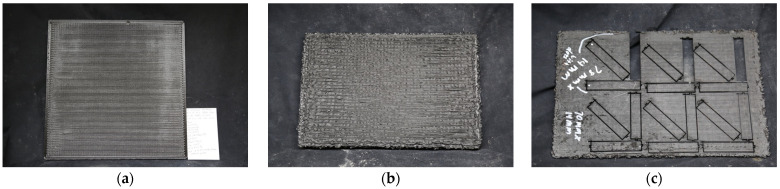
Panel I type and Panel II type: (**a**) Panel I (355.6 mm × 355.6 mm × 8 mm); (**b**) Panel II (300 mm × 196 mm × 20 mm) showing ridging due to over-extrusion; (**c**) planed and waterjet (Panel IIA) with flexural samples in the vertical (90°), diagonal (45°), and horizontal (0°) direction.

Table 1 lists the print settings for the 355.6 mm × 355.6 mm × 8 mm (Panel I) and the 300 mm × 196 mm × 20 mm (Panel II). Panel I type (shown in Figure 2a) was optimized for smooth printed beads and Panel II (shown in Figure 2b) was optimized for least amount of void space, which was achieved through over-extrusion (i.e., increasing the extrusion multiplier to 1.2).

Then, using the settings, Panel I types (for tensile (dried only) and DMA samples (dried and undried)) and Panel II types (for flexural and XRM samples) were printed.

Both panels were decked to approximately 4 mm thickness, except for one Panel II which was kept at the 20 mm thickness for the XRM samples. Samples for DMA, flexural, tensile, and XRM were cut out using a OMAX 5500 water jet (OMAX, Kent, WA, USA) with Barton garnet 80HPA abrasive (Glens Falls, NY, USA). The DMA, flexural, and tensile samples were taken from 0°, 45°, and 90° degrees in the x–y print plane to test the effect of direction, where the x–y plane is the layer where beads are printed and the z-direction refers to the layers stacked on each other. All samples were cut to only contain infill, no perimeter, unless otherwise stated. Figure 2c shows the Panel II with flexural samples in the vertical, diagonal, and horizontal direction. Waterjet samples were allowed to air-dry before testing. Sample edges did not require deburring. None of the samples were annealed and no post-processing besides removing tabbed samples from the panels was conducted. Scrap from undried printed Panels I were used for TGA and DSC testing. Pellets, dried and undried, were also used for TGA testing. Test samples for rheology were injection-molded with a Thermo Scientific MiniJet Pro (Thermo Fischer Scientific, Waltham, MA, USA) at a 250 °C injector temperature and 40 °C mold temperature at a pressure of 350 bar for 30 s. Injection-molded tensile test samples had the same settings as the rheology samples, except the time was 60 s.

### 2.4. Porosity and XRM

**Figure 3:**
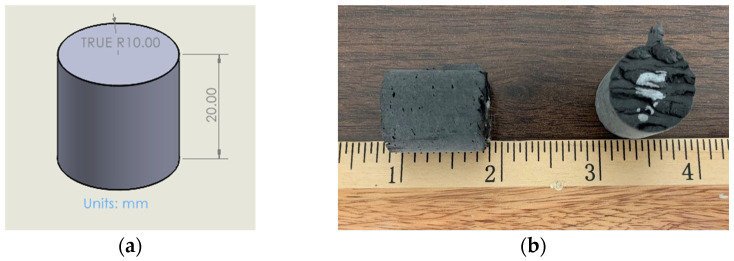
Cylindrical PETG CF30% samples for XRM: (**a**) shape and size; (**b**) printed and removed with ruler in inches.

The porosity of the printed PETG CF30% was studied by taking cylindrical samples from Panel II to view in the XRM. Nine cylindrical samples with a height of 20 mm and an approximate diameter of 20 mm, shown in Figure 3, were taken in a grid pattern of 3 × 3 to achieve a representative porosity across the print. Samples were taken fully from the infill and did not contain any perimeter. The roughness seen at the top of the right sample in Figure 3b was the result of the over-extrusion needed to achieve minimum void space in the print. The sample was not decked and included material from the top and bottom of the print.

The samples were mounted on a wooden platform to minimize artifacts in the scans caused by attenuation differences of mounting. The platform was held in place using a pin vice. Samples were scanned using a ZEISS Xradia 620 Versa X-ray Microscope (ZEISS, Oberkochen, Germany) and a 0.4× objective to allow for a full field three-dimensional digital reconstruction of the samples. The distance between the source and objective was held constant to allow for all nine reconstructions to have a consistent pixel length of 39.2 μm. The voltages, current, and temperature were held constant to mitigate difference between scans. Due to the homogeneity of the samples, standard grayscale methods do not allow a detailed three-dimensional visualization. Image processing was done with the Dragonfly Pro (ZEISS, Oberkochen, Germany) software (version 2021.1) developed by Object Research Systems (ORS) to apply lighting and shadows to the structure to provide details in a three-dimensional environment. Dragonfly software was used to apply Otsu’s method. Small artifacts (image distortion) were present due to the low voltage resulting in noise, small sample drift, thermal shift, beam hardening, and streak artifacts due to attenuation differences. A cylinder of height 17 mm and a diameter of 18 mm was taken from each digital reconstruction to reduce the number of artifacts while still having a representative volume (equivalent to 59.35% of the sample’s original volume). Samples were considered to be of uniform composition.

### 2.5. DSC

DSC was used to determine the T_g_ of the PETG CF30% from the undried Panel I. Three samples were taken from the pelletized material, which had been exposed to ambient air, and three samples were taken from a printed undried panel, from approximately the same area of the panel. The sample was heated to 340 °C to remove any residual thermal history, cooled to −90 °C, and then heated to 340 °C, with a heating and cooling rate of 10 °C/min. T_g_ was taken from the second heating curve to consider material properties. All samples were tested on a TA Instruments DSC 250 (TA Instruments, New Castle, DE, USA), and all samples were sealed in Tzero Aluminum Pans (TA Instruments, New Castle, DE, USA) with a Tzero Hermetic Lid (TA Instruments, New Castle, DE, USA). TRIOS software was used to calculate the glass transition temperature (T_g_).

### 2.6. DMA

All testing was completed on a TA Instruments RSAG2 (TA Instruments, New Castle, DE, USA) with an air-chilled cooler (ACS-2) using the three-point bending fixture. The samples were 50 mm × 14 mm × 4 mm, adjusted according to ASTM D7028 [22] to fit the testing equipment. Three DMA samples from undried Panel I from the vertical, horizontal, and diagonal direction were tested. A dynamic temperature ramp was conducted from −50 °C to 200 °C at a ramp of 3 °C/min and an initial soak time of 60 s. The test was conducted at 1% strain and a frequency of 1 Hz. Three DMA samples from a dried Panel I from the vertical, horizontal, and diagonal direction were tested. Temperature sweeps were conducted from 25 to 120 °C at 3 °C/min. One DMA sample from a dried Panel II from the vertical, horizontal, and diagonal direction was tested. Temperature sweeps were conducted from room temperature (~25 °C) to 120 °C at 3 °C/min.

### 2.7. Rheology

Rheology was conducted on a TA Instruments DHR-2 (TA Instruments, New Castle, DE, USA) using parallel plates and environmental testing chamber. Injection-molded discs of PETG CF30% with a diameter of 25 mm and thickness of 2 mm were tested. The test gap was set to 2 mm. Viscoelastic frequency sweeps were conducted at temperatures of 180–320 °C with frequency range of 0.1–100 rad/s at a constant amplitude of 1% strain.

### 2.8. Flexural Testing

The flexural samples were cut from two separate Panel II prints, with one print labeled as Panel IIA and the other labeled as Panel IIB. Panel IIA had samples of 78 mm × 14 mm × 4 mm, with some horizontal samples containing perimeter and infill. For Panel IIB, one set of samples in three directions was 78 mm × 14 mm × 4 mm and the rest were 70 mm × 14 mm × 4 mm, with some horizontal samples containing perimeter and infill. The 78 mm × 14 mm × 4 mm sample size was calculated based on ASTM D790-17 [23] and testing equipment size limitations. The data were validated by calculating the stress from the recorded force value using the equation provided in ASTM D790-17 and basic mechanics of materials (beam with a pin and a roller).

Samples were tested using ASTM D790 Procedure A [23]. A minimum of four samples were tested from each panel and in each orientation (horizontal, vertical, and diagonal) relative to the x–y plane, as shown in Figure 2c of Section 2.3. The support span for Panel IIA samples was calculated with a span-to-thickness ratio of 16:1. Because the majority of Panel IIB samples were shorter, a ratio of 9:1 or 10:1 was used for Panel II samples, including the 78 mm × 14 mm × 4 mm samples. All samples were tested at a uniform displacement rate of 2.5 mm/min. A preload of 5–15 lbf was applied to the sample prior to testing. Bluehill Universal software (version 4.42) was used to record and analyze the data obtained. An Instron 5985 (Instron, Norwood, MA, USA) was used for testing.

### 2.9. Tensile Testing

**Figure 4:**
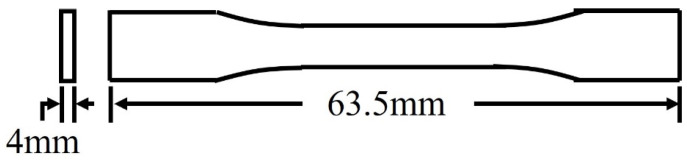
Tensile sample size and shape from ASTM D638-14 Type V.

An Instron Electropuls E3000 (Instron, Norwood, MA, USA) with environment control chamber was used to test PETG CF30% tensile samples, with samples cut in three directions (horizontal, vertical, and diagonal) and injection-molded tensile samples. Tests were executed at room temperature (RT), 40, 50, 60, 70, and 80 °C to provide information on how the material’s strength properties (ultimate tensile strength and Young’s modulus (E)) change as the temperature changes. The displacement rate was 2 mm/min. Bluehill Universal software (version 4.25) was used to record and analyze the data obtained. At least four samples were run for each experiment. Tensile samples were ASTM D638-14 [24] Type V, as seen in Figure 4, with a thickness of 4 mm to provide two layers of printed bead. Full dimensions can be found in ASTM D638-14 [24].

## 3. Results and Discussion

### 3.1. TGA to Determine CF Content of PETG

**Figure 5:**
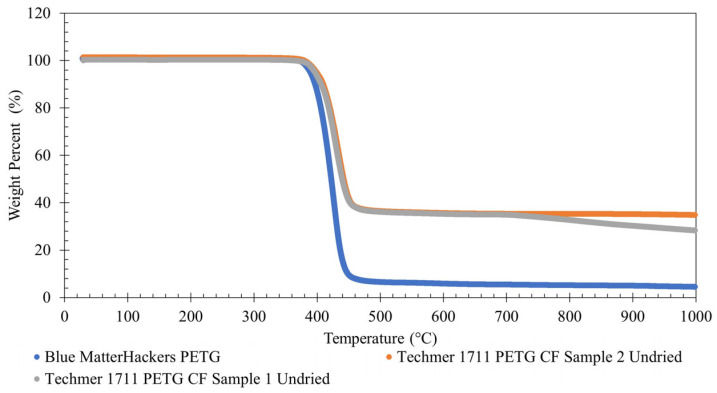
TGA comparison of weight percentage for PETG CF and PETG.

Figure 5 shows the onset degradation temperature and weight remaining at 600 °C for PETG CF and MatterHackers Blue PETG. PETG CF Sample 1 shows a steady decrease after about 700 °C that PETG CF Sample 2 does not show. Thermal degradation corresponds to weight loss because, at elevated temperatures, rupture or scission of molecular chains can occur [16].

Table 2 shows the weight percentages and onset degradation temperature for all three materials. If the 6% is assumed to be residual weight of PETG at 600 °C, then the resulting leftover CF weight percentage is 29% for Sample 1 and 30% for Sample 2.

All three materials have a degradation onset temperature around 400 °C, with the PETG CF30% having a somewhat higher degradation onset temperature. For comparison, Quintana et al. (2022) determined neat PETG (Techmer Hifill PETG 17043DP, Techmer PM, Clinton, TN, USA) to have an onset degradation temperature of 387.61 ± 1.68 °C [6], and Huseynov, Hasanov, and Fidan (2023) found that neat PETG (Push Plastics, Springdale, Arkansas) had an onset degradation temperature of 405 °C and PETG CF8% had an onset degradation temperature of 398.5 °C [25]. The disparity in degradation temperature between Quintana et al. (2022) and the current work is assumed to be due to (1) addition of carbon fiber and (2) possible difference in PETG type or other additives (Techmer Hifill PETG 17043DP vs. Techmer Electrafil PETG 17113DP), while the disparity with Huseynov, Hasanov, and Fidan (2023) is assumed to be due to difference in PETG type. Given that PETG CF30% starts to degrade at 400 °C, PETG CF30% should not be printed at 400 °C or higher.

### 3.2. Porosity and XRM

**Figure 6:**
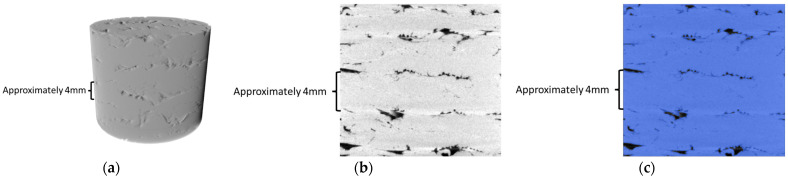
XRM Sample: (**a**) 3D digital reconstruction of PETG CF 30% sample from XRM projections; (**b**) two-dimensional slice of digital reconstruction before segmentation; and (**c**) after segmenting material (blue) from surrounding air.

The inter-bead void space (void space between the beads) occurs naturally in the 3D printing process [3] and can be limited by increasing the extrusion multiplier [26] or over-extruding. Figure 6a shows the resulting three-dimensional reconstruction of a segmented sample from the XRM from Panels II. The layers are generally noticeable, given the layered sections of void space; however, no individual bead is visible. More specifically, the void space is limited and occurs mainly between layers in the z-direction rather than the x or y. Due to over-extrusion, the bead was squished into the one next to it during printing, eliminating the void space. Figure 6b shows a two-dimensional slice taken from the coronal plane of the sample. Due to the contrast difference, image processing software can allow thresholding to accurately segment the material from the air present inside. Figure 6c shows the isolation of the material in blue from the air present in the sample. The nominal layer height was 2 mm; however, over-extrusion resulted in layer heights for the middle layers that were approximately 3.5–4 mm.

Once the material is segmented from the surrounding air, Dragonfly software allows for the automatic calculation of the labeled material pixel total of every projection in the reconstruction. The porosity can then be calculated using Equation (1), using the porosity, φ, volume of the material, V_m_, and the total volume of the sample, V_t_ [27]. In this instance, the total volume will correspond to the volume of the cylinder taken from the original digital reconstruction.

(1) $$\varphi =\frac{{(V}_{t}-V_{m})}{V_{t}}$$

The results of the segmentation and corresponding porosity of each sample are shown in Table 3. The XRM samples had an average porosity of 1.63%, which indicates that most of the sample is matrix material with a small concentration of voids present. The porosity percentage has a standard deviation of 0.179% and a coefficient of variance of 0.11%, which indicates good consistency across samples.

Decreased void space can be achieved by over-extrusion, which decreases the dimensional accuracy of a PETG CF30% printed final part. Further research can consider the relative bead-to-inter-bead void space of desktop and LFAM prints and the effect of print settings on the strength of LFAM, focusing on the effect of void space.

### 3.3. DSC Results

**Figure 7:**
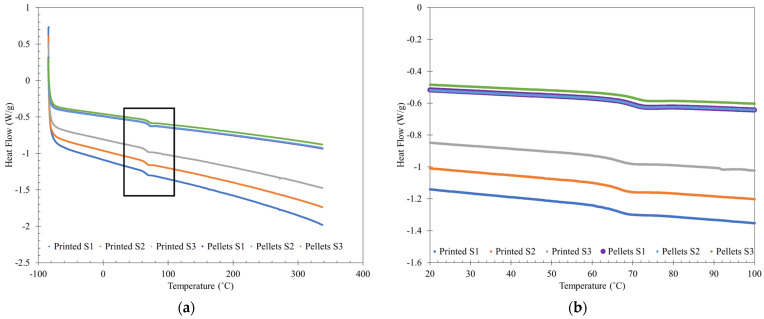
DSC curve of undried pellets and printed PETG CF30% from undried pellets: (**a**) entire DSC curve; (**b**) close-up of T_g_ curve.

Figure 7 shows the DSC curve of undried pellets and printed material from undried pellets, with T_g_ curve in Figure 7a denoted by the black box and where S in the legend denotes “Sample”. In both Figure 7a,b, the inflection between 60 °C and 70 °C indicates the T_g_. In Figure 7b, Pellets S1 (purple) and Pellets S2 (light blue) have the same T_g_ and curve shape.

PETG is an amorphous material, so it does not have a melting temperature [4,6]. As expected, none of the tested samples exhibit a crystallization peak or a melting peak. The T_g_ occurs at approximately the same temperature, indicating that printing the material had limited effect on the T_g_. Table 4 shows the T_g_ values for PETG CF30% for undried pellets and a print from undried pellets. The minor differences in T_g_ between pellets and printed could be caused by hydrolysis that occurs during the printing process of wet pellets, shortening the polymer chains [28] and resulting in a slightly lower T_g_ [16] for the printed samples. Huseynov, Hasanov, and Fidan (2023) found that PETG CF8% had a T_g_ of 70.10 °C, though whether the tested sample was filament- or 3D-printed was not specified [25]. The difference in T_g_ could be a difference in PETG brand and is relatively insignificant.

Printing PETG CF30% from undried pellets has a small but limited effect on the T_g_, decreasing the T_g_ by a couple of degrees. Processing the undried PETG CF30% by LFAM does not affect the overall thermal behavior of PETG CF30%.

### 3.4. DMA Results

DMA testing was executed to determine the T_g_ across three different directions for three different print and material settings. Table 5 shows the average T_g_ values for undried printed PETG CF30% and dried printed PETG CF30% taken from Panel I panels and a single T_g_ value for dried printed PETG CF30% taken from Panel II. Each had samples taken from the vertical, horizontal, and diagonal directions. All T_g_ values were calculated using the tan delta method.

The T_g_ is not greatly affected by sample direction, manufacture choice, or pellet dryness. For comparison, Quintana et al. list PETG as having a T_g_ of about 77.5 °C [6] and Blok et al. (2020) have PETG at a range of 81–91 °C [4]. Bhandari et al. (2019) found that the T_g_ of PETG, using the peak tan delta method, was 90.2 °C [29]. Using the peak tan delta method, Subbarao et al. (2020) found PETG printed at three different layer heights (0.17 mm, 0.23 mm, 0.3 mm) resulted in different T_g_ temperatures (79 °C, 76 °C, 76 °C, respectively) [30]. Quintana et al. (2022), using DSC for measurement, found that adding PETG with glass fiber (30% wt.) and polycarbonate (PC) with CF (25% wt.) decreased T_g_ of the composite by ~1.5 °C and ~4 °C, respectively, but ABS with CF (20% wt.) increased T_g_ of the composite by ~2 °C when comparing with the neat material [6], indicating that the addition of CF had likely affected the T_g_ of PETG CF30% overall. Holcomb et al. (2022) found that PETG CF had a higher T_g_ than PETG but did not explicitly report the T_g_. Estimations from the graphs suggest that the T_g_ for PETG was ~80 °C and the T_g_ for PETG CF was ~82.5 °C [31]. In conclusion, the data reported here are comparable to those in the literature.

**Figure 8:**
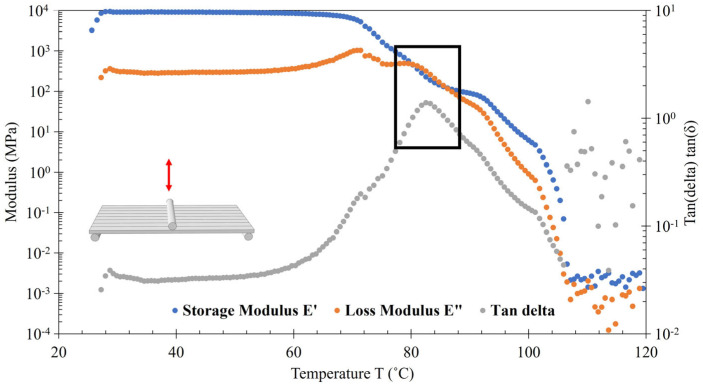
DMA measurement of PETG CF30% vertical, printed, dried. Arrow indicates repeated cyclical motion of DMA testing on 3-point bending test.

A representative DMA graph, Figure 8, shows the storage modulus (E′), loss modulus (E″), and tan delta for the vertical direction of PETG CF30% printed, dried. T_g_ was found using the peak of the tan delta (gray). Initially, both the storage modulus (E′, blue) and the loss modulus (E″, orange) remain approximately steady from room temperature until around 50 °C. The tan delta curve steady increases throughout, first with a low slope, and then increases to a greater slope, having a slight peak around 70 °C, reflecting the loss modulus peak and then reaching a maximum peak around 80 °C. The storage modulus and the loss modulus cross over around 80 °C. This corresponds with the peak of tan delta, which is used to determine the T_g_. The loss modulus crosses back below the storage modulus. Then, as temperature continues to increase, the loss and storage modulus and tan delta drop until the data become sporadic around 110 °C. The sporadic data points reflect where the sample loses contact with the fixture due to the sample warping above T_g_.

### 3.5. Rheology Results

**Figure 9:**
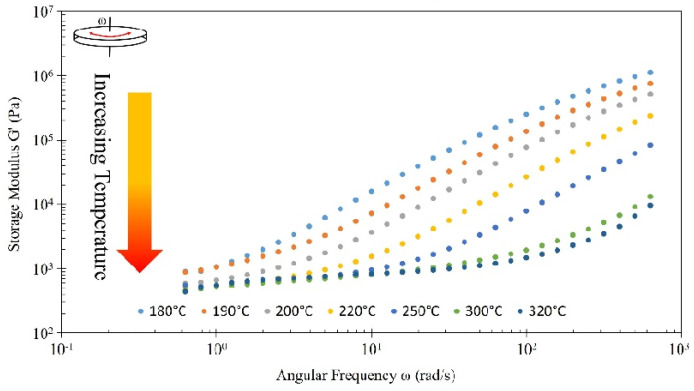
Frequency sweep of PETG-CF30% for temperatures ranging from 180 to 320 °C. Sample testing temperature generally increases in direction of arrow, with a visual representation of yellow indicating warm temperatures and red indicating hotter temperatures. Provided legend also indicates sample testing temperature.

Rheological behavior for PETG CF30% for a range of frequencies at different temperatures (180 °C, 190 °C, 200 °C, 220 °C, 250 °C, 300 °C, 320 °C) is shown in Figure 9. For all temperatures studied, G″ (viscous component) response was higher than G′ (elastic component). As temperature is increased, the G′ responses overall are lowered in magnitude, indicating lower viscoelasticity at elevated temperatures. All temperatures tested are in the melt regime and do not show terminal behavior. The absence of terminal behavior at low frequencies is likely due to the loading of the CF within the material. Terminal behavior would be observed by having a constant slope throughout the tested frequency range. The data seem to converge with similar plateaus at low frequency.

**Figure 10:**
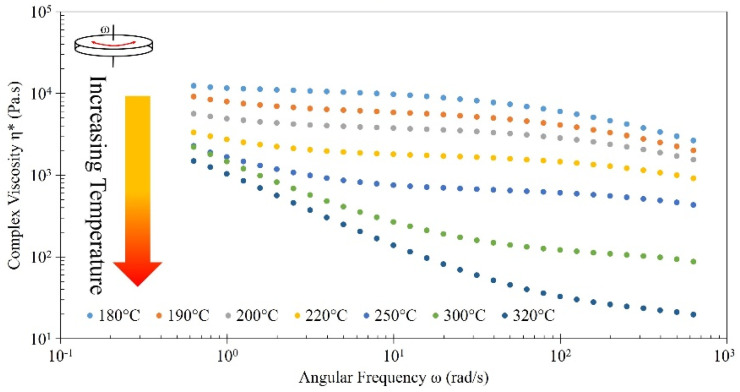
Complex viscosity as a function of frequency for PETG CF30% from 180 to 320 °C. Sample testing temperature generally increases in direction of arrow, with a visual representation of yellow indicating warm temperatures and red indicating hotter temperatures. Provided legend also indicates sample testing temperature.

Figure 10 shows the relationship between angular frequency and complex viscosity for all tested temperatures. At moderate temperatures (180–200 °C), there are Newtonian features at lower frequencies, and some slight shear thinning at higher frequencies is observed. Around printing temperatures (220–250 °C), slight shear thinning occurs at low frequencies, a plateau occurs at mid-frequency, and thins slightly again at high frequencies. The printing temperature used was 250 °C, making the 220–250 °C range of interest, as it is most reflective of what is occurring in the nozzle during extrusion. At higher temperatures (300–320 °C), the material is high in shear thinning at lower frequencies, with 300 °C plateauing at higher frequencies and 320 °C continuing to exhibit shear-thinning behavior, though at a lesser degree. Arrigo and Frache 2022 note that prominent shear-thinning behavior is considered beneficial in nozzle extrusion in FDM printers with filament extrusion because it decreases the pressure needed for extrusion [32]. Because PETG CF30% exhibits some shear thinning, it has proven sufficient for good extrusion from a screw extruder.

### 3.6. Flexural Results

Flexural testing based on ASTM D790-17 procedure A (three-point bend) was conducted to determine the flexural strength and flexural modulus for Panels II, with samples taken from the horizontal, vertical, and diagonal direction. Samples were laid flat-wise, as specified by the standard. Samples had a rectangular cross-section of 56 mm^2^. Flexural samples were taken from two panels, referred to as Panel IIA and Panel IIB. Data from Panel IIA in the diagonal direction were lost and are not included in this analysis but were visually consistent with the unanalyzed results from Panel IIB in the diagonal direction. For the vertical samples from Panel IIB, Samples 1–4 matched the calculated stress when the recorded span was almost doubled. Sample 5 matched the calculated stress with the recorded span. New stress values for Samples 1–4 were calculated using the equation provided in ASTM D790-17 using the recorded span. Table 6 shows the average flexural modulus and average flexural strength for horizontal, vertical, and diagonal samples from Panel IIA and Panel IIB. The span-to-support depth-testing ratio is listed next to the direction.

All flexural strength is within the same magnitude. Kichloo et al. (2022) found that prints on a desktop 3D printer with a variety of infill types (grid, triangular, and honeycomb), infill percentages (40%, 70%, and 100%), and layer heights (0.1 mm, 0.14 mm, and 0.18 mm) had a range of flexural strength ~30–68 MPa for PETG and ~38–72 MPa for PETG CF20% [2]. The average flexural strengths for all samples fall within the range from Kichloo et al. (2022), which were 3D-printed. Techmer lists the flexural modulus as 16.2 GPa and the flexural strength as 138 MPa for injection-molded test samples [33], which is greater than the reported average flexural modulus and average flexural strength. This reflects the fact that 3D-printed samples are weaker than traditionally manufactured samples [3]. The data indicate that the material processing (3D printing and print parameters) has a profound effect on strength, even with the additional carbon fiber.

Table 7 shows the average, minimum, and maximum flexural strength values. There is significant variability within the sample data. Several factors are likely causing the flexural strength variability. Generally, in FFF printing, there is variability between parts of the same type and variability across a single print [34]. Void volume varies across the print, as seen in Figure 6, as a result of the print process. Gurrala and Regalla (2014) also found variation in neck size (implying variation in void space) in FFF-printed ABS [35]. Some horizontal samples with 16:1 and 10:1 ratios included some of the perimeter instead of just infill. Additionally, samples had numerous surface defects from decking revealing the inter-bead void space, with the void space ranging from small divots to sections where the cross-sectional area was reduced by as much as 40%. Samples with large surface defects were prone to breaking to the left or right of the centerline of the sample because higher stress concentrations occurred due to sharp edges where material was not uniformly present. One set (horizontal, vertical, and diagonal) of 78 mm × 14 mm × 10 mm samples was in panel IIB; however, the difference in overhang is not expected to have any major effect on reported material strength because the stress-strain calculations are based on support span length [24,36].

### 3.7. Tensile Results

**Figure 11:**
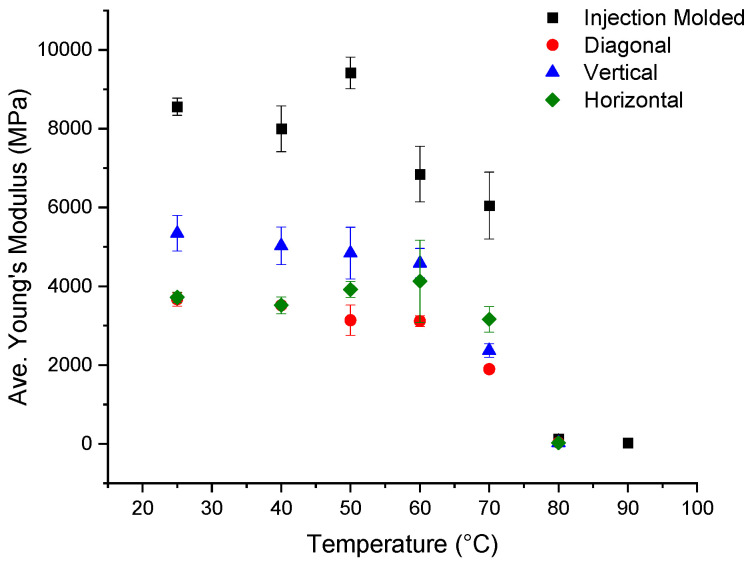
Summary of average Young’s-modulus values of injection-molded samples and the 3D-printed samples in the vertical, horizontal, and diagonal directions.

Tensile samples from the dried Panel I and injection-molded samples were tested at different temperature ranges for Young’s modulus and tensile strength. Figure 11 summarizes the average Young’s modulus values for each direction compared with the injection-molded samples. Injection-molded samples exhibited higher modulus values than the 3D-printed samples, until around the T_g_, where the Young’s modulus decreased rapidly. A sudden change in Young’s modulus at T_g_ is expected [16] because above the T_g_, amorphous polymers, such as PETG, soften and lack structural use [4].The injection-molded samples have a greater average Young’s modulus by 67.4% at room temperature and by 131.5% at 80 °C, on average, than the 3D-printed samples in all directions.

**Figure 12:**
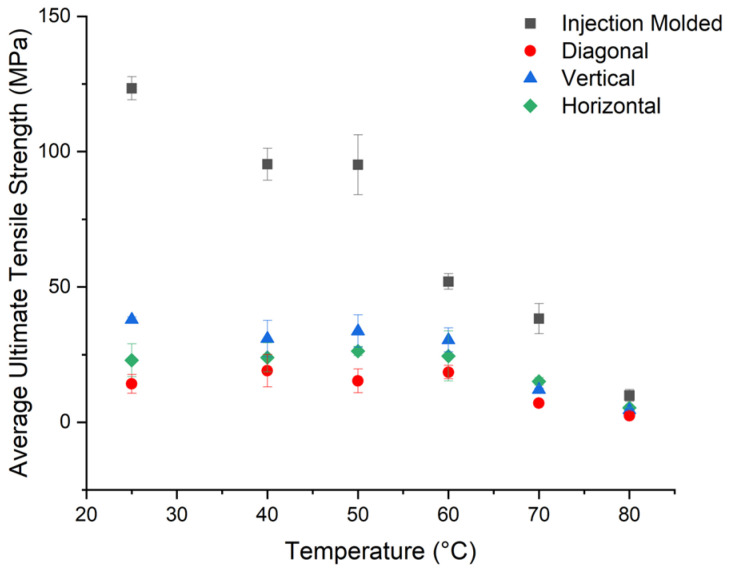
Summary of average ultimate tensile strength values of injection-molded samples and the three different directions.

Figure 12 shows the average ultimate tensile strength of PETG CF30% for injection-molded and 3D-printed samples. The 3D-printed samples came from Panel I, printed with dried PETG CF30%. The injection-molded samples are significantly stronger, especially at 50 °C or less. As the temperature approaches the T_g_, the strength decreases significantly for the injection-molded samples, and at T_g_, all samples have a similar strength. Samples 3D-printed are expected to be weaker than injection-molded samples at room temperature, as 3D printing creates voids within the system, which can serve as crack initiators and areas of stress concentrations [3]. The decrease in strength around T_g_ reflects the decrease in Young’s modulus seen in Figure 11. The decrease in strength is expected as the material switches from rigid to rubbery [16]. The injection-molded samples have a greater average ultimate tensile strength by 132.6% at room temperature and by 82.8% at 80 °C than the 3D-printed samples in all directions. Appendix A contains representative tensile graphs for vertical, diagonal, and horizontal samples.

While the 3D-printing process orients the carbon fiber in the direction of printing [6,21], thereby increasing strength in the printing direction [21], the panels in this work were printed in an alternating 0°/90° direction, mitigating most of the anisotropy in the x–y print plane. The samples were decked to a thickness of 4 mm and would include, roughly, the equivalent of one layer in the 0° direction and one layer in the 90° direction (often a full layer sandwiched by two half-layers), which provided roughly equal strength in the x and y direction of the print plane.

### 3.8. PETG and PETG CF Ultimate Tensile Strength Comparison

**Figure 13:**
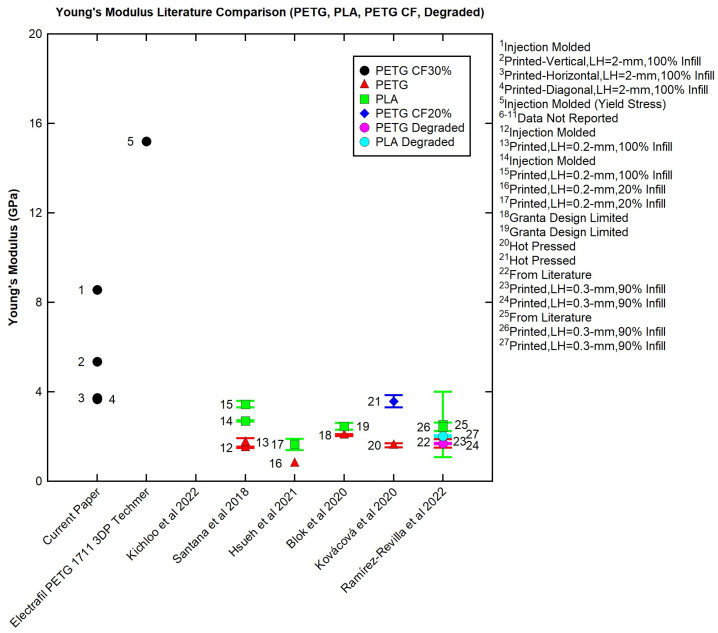
Young’s-modulus comparison of PETG, PLA, PETG CF, and degraded PETG and PLA with values from the literature [2,4,5,9,33,37,38].

**Figure 14:**
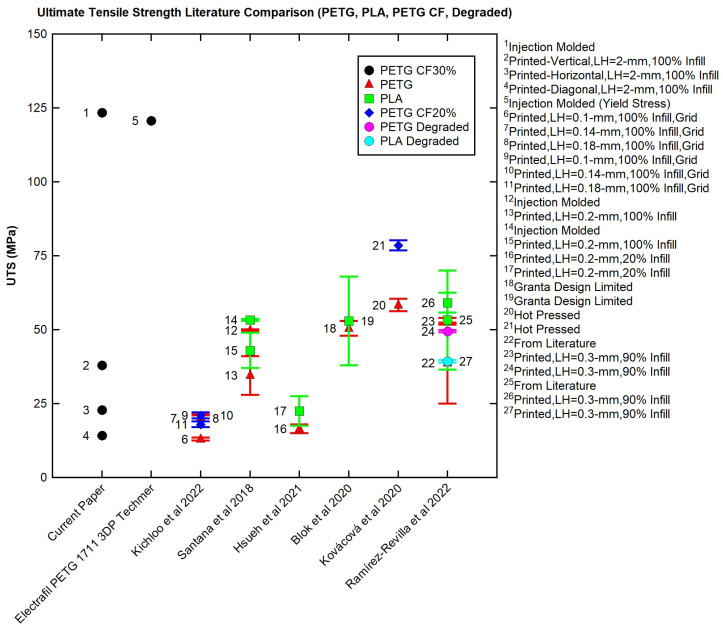
Ultimate tensile strength comparison of PETG, PLA, PETG CF, and degraded PETG and PLA with values from the literature [2,4,5,9,33,37,38].

The room temperature tensile values were compared to the tensile results found in the literature for PETG and PETG CF. The Young’s-modulus values are graphed in Figure 13 and the ultimate tensile strength values are graphed in Figure 14 for comparison. Note that the long error bars indicate ranges from multiple sources or a variety of tests. The shorter error bars refer to error for one set of tests.

Kichloo et al. (2022) did not report a Young’s modulus, as denoted by the lack of data in Figure 13. Injection-molded PETG CF30% from the current paper (point 1) and from Techmer (point 5) have the two highest Young’s moduli. Vertical PETG CF30% (point 2) has the third highest Young’s modulus, indicating that the 3D-printing process decreases the Young’s modulus but does not negate the effect of the material strength. Horizontal (point 3) and diagonal PETG CF30% (point 4) have very similar Young’s-modulus values and are comparable to the Young’s modulus of hot-pressed PETG CF20% from Kováčová et al. (2020) (point 21) as well as 3D-printed PLA from Santana et al. (2018) (point 15) and the upper bound of the PLA data from Ramírez-Revilla (point 25). Use of 3D printing results in a decreased Young’s modulus; however, material choice has a strong impact on the Young’s modulus, with vertical PETG CF30% (point 2) having a greater Young’s modulus when compared to all values, excluding points 1 and 5, and the diagonal and horizontal PETG CF30% (points 3 and 4) having a Young’s modulus at the upper bounds of the compared values, excluding points 1, 2, and 5.

The printed PETG CF30% (points 2–4) had ultimate tensile strength in the middle or lower end of the 3D-print comparisons and significantly less than the injection-molded samples (point 1 or point 5 (yield stress)). Several factors affect the final strength and should be considered when comparing tensile strength: material choice, sample creation process, and print parameters if printed. Samples made with CF tended to have higher strength than their neat comparison (Kichloo et al. 2022 and Kováčová et al. 2020). In regard to sample manufacture, samples created using injection molding or a non-3D-printing process (points 1,5,12,14,20,21) would lack the void space, stress concentrations, and limited polymer entanglement that decrease the strength of 3D prints [3,4,12,13], generally making them stronger. Decking and then waterjetting the PETG CF30% samples likely impacted strength by removing material at the top and bottom of the print, opening additional stress concentrations by exposing void space from the 3D-printing process along the side of the printed sample, and reducing the integrity of the part overall. Additionally, the 2 mm layer height for the current work may have been small enough to introduce significant stress concentrations (large voids) not seen in smaller prints but too small to benefit from a larger printed bead serving as a continuous part for the sample. In regard to print parameters, the number of perimeters can affect final tensile strength and, depending on print parameters, can provide strength in the direction of loading for tensile testing [39]. Desktop 3D-printed samples either explicitly listed perimeters or the infill required perimeters [2,5,37,38]. For the current work, the samples were cut from the infill and did not have any perimeters to provide strength in the direction of loading. Additionally, PETG CF30% may not be able to fully capitalize on the material strength due to the printing method and print parameters selected. As seen in Kichloo et al. (2022), poorly chosen print parameters can negate the effect of added carbon fiber [2]. Finally, if the print is large and the layer below cooled significantly, limited polymer chain entanglement may have occurred, harming the strength.

When a material is selected for LFAM, the material strength must be considered in conjunction with the print parameters. While LFAM PETG CF30% had a high Young’s modulus compared to the compared values found in the literature, LFAM PETG CF30% had a low tensile strength when compared to the values found in the literature. The process (LFAM) has a large impact on the part structure, which impacts the overall part strength. For desktop printing, the printed beads are much smaller, which would allow for less void space, more packing, and depending on print settings and related heat transfer and polymer chain mobility, potentially better bonding between layers. As LFAM becomes more common, materials testing on LFAM parts will become more prominent. LFAM at a 2 mm layer height may not be directly comparable to LFAM at a 5 mm layer height. A potential way to view 3D printing is the idea of the bead–layer–part structure, which is analogous to micro–meso–macro. Micro–meso–macro is not very accurate at the mm length scale, where LFAM prints. A test sample, its results, and the interpretation of the results would differ depending on the printer it came from—a desktop printer (part), a smaller LFAM printer (layer), or a large LFAM printer (bead)—as well as the material used and the print parameters. Creating a more complete understanding of the relationship between printing and properties and considering how that relationship scales is needed for successful implementation of LFAM printing.

## 4. Conclusions

This article determined the thermomechanical properties of PETG CF30%, LFAM-printed (2 mm layer height) and unprinted.

- TGA determined that the degradation temperature was ~405 °C. The carbon fiber content was 30%.
- Over-extrusion of dried PETG CF30% in Panel II had an average void space of 1.63%.
- DSC determined that undried PETG CF30% pellets (~69 °C) and printed undried PETG CF30% (~65 °C) had similar T_g_ values.
- The DMA results showed that T_g_ was not greatly affected by material drying, panel type (I vs. II), or the direction that the panel was taken from (horizontal, vertical, or diagonal).
- The results shown in Table 5 demonstrate that the direction from which a printed sample is taken has little effect on T_g_, indicating that the thermal properties across the sample are very similar. There is little effect based on directionality.
- PETG CF30% exhibits shear thinning around printing temperature (220–250 °C), a good quality for a printing feedstock.
- All flexural samples, regardless of direction, showed a decreased flexural strength and flexural modulus when compared to the data in the Techmer data sheet. The data variability is likely due to void space variation and location on the print (infill vs. perimeter).
- There was a decrease in Young’s modulus and strength for 3D-printed and injection-molded samples when T_g_ was reached. Injection-molded samples have greater tensile strength across all temperatures when compared to 3D-printed samples. Injection-molded samples have greater tensile modulus until 80 °C.
- For the literature comparison, injection-molded PETG CF30% has the greatest tensile strength. PETG CF30% 3D-printed in all directions has similar or less tensile strength when compared to PETG printed on desktop printers. The difference is likely due to cutting the samples out of the panel, which opened stress concentrations on the edge of the sample, the lack of printed perimeter, and differences in scaling between desktop and mid-sized LFAM printing.
- For LFAM, material and print parameters must be considered. Additionally, bead–layer–print structure should be used to compare LFAM data. Finally, testing method affects reported results.

Future work should consider the scaling effect across a larger range of bead sizes (0.1–5 mm), especially the effect of packing of printed beads and the difference in stress concentrations across void space size. The difference in stress concentrations on sample edges between 2 mm layer height and desktop tensile samples printed with and without perimeter should also be considered.

## Figures and Tables

**Table 1:** Print parameters for panels.

Print Parameter	Panel I	Panel II
Material	PETG CF30%	PETG CF30%
Drying State	Undried, Dried	Dried
Nozzle Diameter (mm)	3	3
Nozzle Temperature (°C) (PID)	250	250
Print Bed Temperature (°C) (PID)	80	80
M3 Motor (Mach4) (counts/unit)	276	276
Nozzle (on printer)	3 mm	3 mm
Layer Height (mm)	2	2
Initial Layer Height (mm)	2	2
Maximum Volumetric Extrusion (mm^3^/s)	500	500
Maximum Print Speed (mm/s)	500	500
Infill Print Speed (mm/s)	600	600
(In)Fill Pattern	Rectilinear	Rectilinear
(In)Fill Angle	0/90	0/90
Infill Percentage (%)	100	100
Infill–Perimeter Overlap (%)	20	20
Filament Diameter (mm)	5.5	5
Nozzle Diameter (mm)	3	3
Extrusion Multiplier	1	1.2
Perimeter (beads)	3	3
Agitator	Undried: hand tapped Dried: 3 off 1 on	3 off 1 on

**Table 2:** TGA data for weight % remaining at 600 °C and degradation onset temperature.

Material (Tested in Nitrogen)	Weight Percentage Remaining at 600 °C (%)	Degradation Onset Temperature (°C)
PETG CF Undried Sample 1	35	405.91
PETG CF Undried Sample 2	36	407.35
MatterHackers PETG Blue Filament	6	400.19

**Table 3:** Porosity by XRM sample from Panel II type, printed with dry PETG CF30%.

Sample	Volume of Material (V_m_) (mm^3^)	Porosity (φ) (%)
1	4262.38	1.47
2	4263.68	1.44
3	4244.64	1.88
4	4268.01	1.34
5	4259.35	1.54
6	4251.57	1.72
7	4256.76	1.60
8	4265.41	1.40
9	4226.91	2.29
Average	4255.41	1.63

**Table 4:** T_g_ Data for pellets and printed PETG CF30% using the DSC.

Sample	T_g_ in °C, Pellets	T_g_ in °C, Printed
1	68.17	65.21
2	68.89	65.07
3	69.84	65.07
Average	68.97	65.12

**Table 5:** T_g_ talues of PETG CF30% in three print conditions in the vertical, horizontal, and diagonal directions.

Orientation	Undried Panel I (°C)	Dried Panel I (°C)	Dried Panel II (°C)
Vertical	82.10	81.85	82.15
Horizontal	82.79	82.33	82.31
Diagonal	82.44	82.65	81.47

**Table 6:** Average flexural modulus and average maximum flexural strength for horizontal, vertical, and diagonal samples from Panel IIA and IIB.

Panel	Direction/Sample Type (Ratio)	Average Flexural Modulus (GPa)	Std. Dev.	Average Maximum Flexural Strength (MPa)	Std. Dev.	Number of Samples Tested
IIA	Horizontal (16:1)	3.66	0.42	44.78	17.97	4
	Vertical (16:1)	3.23	0.57	53.18	7.78	4
IIB	Horizontal (10:1)	2.43	0.53	48.92	16.29	5
	Vertical (10:1)	n/a	n/a	68.68	19.46	5
	Diagonal (9:1)	1.95	0.35	47.69	9.35	6

**Table 7:** Minimum, average, and maximum of maximum flexural strength for horizontal, vertical, and diagonal samples from Panel IIA and IIB.

Panel	Direction/Sample Type (Ratio)	Maximum Flexural Strength (MPa)		
		Minimum	Average	Maximum
IIA	Horizontal (16:1)	25.56	44.78	66.17
	Vertical (16:1)	45.11	53.18	63.63
IIB	Horizontal (10:1)	30.87	53.44	65.78
	Vertical (10:1)	46.96	68.68	93.25
	Diagonal (9:1)	35.86	47.69	59.38

**Figure A1:**
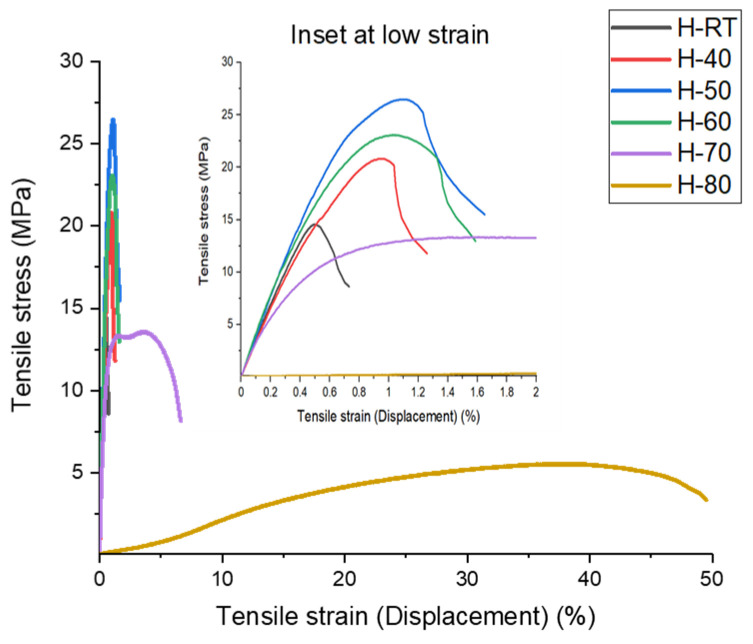
Representative Tensile Data for 3D-Printed PETG CF30% Sample Taken from the Horizontal Direction.

**Figure A2:**
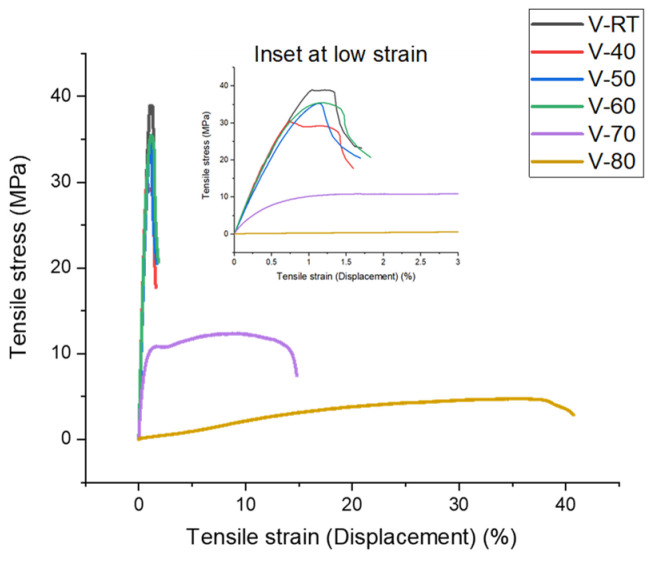
Representative Tensile Data for 3D-Printed PETG CF30% Sample Taken from the Vertical Direction.

**Figure A3:**
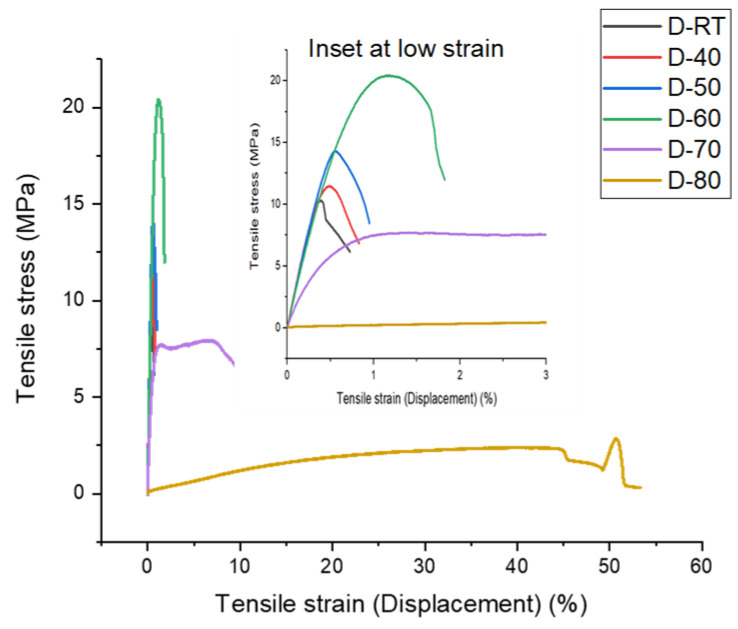
Representative Tensile Data for 3D = Printed PETG CF30% Sample Taken from the Diagonal Direction.

## Data Availability

The original contributions presented in the study are included in the article. Further inquiries can be directed to the corresponding author.

## Appendix A. PETG CF30% Tensile Graphs

Figure A1, Figure A2 and Figure A3 are the representative tensile graphs for PETG CF30%, with the samples taken from the horizontal, vertical, and diagonal direction in the x–y printed plane. The numbers in the legend refer to the environmental temperature at time of testing (i.e., RT refers to room temperature and 60 refers to 60 °C), and the letters refer to the direction (i.e., H refers to horizontal).
